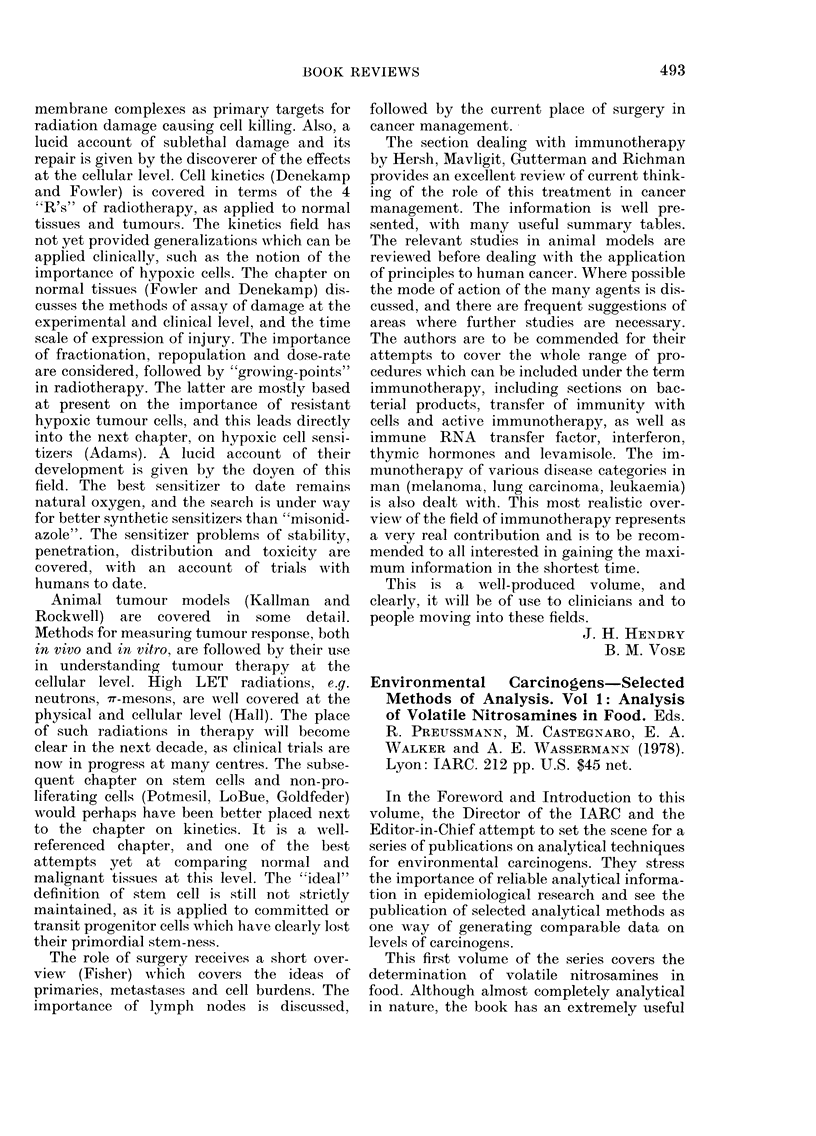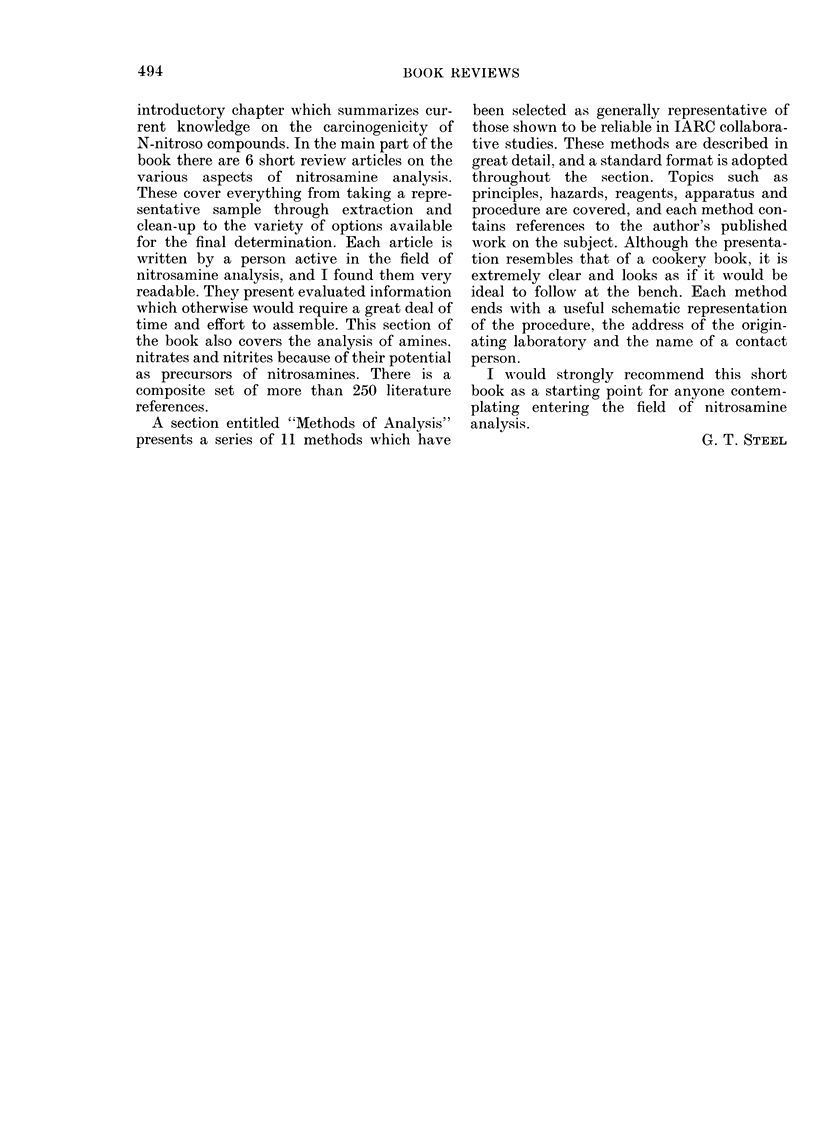# Environmental Carcinogens—Selected Methods of Analysis. Vol 1: Analysis of Volatile Nitrosamines in Food

**Published:** 1979-04

**Authors:** G. T. Steel


**Environmental Carcinogens—Selected Methods of Analysis. Vol 1: Analysis of Volatile Nitrosamines in Food.** Eds. R. Preussmann, M. Castegnaro, E. A. Walker and A. E. Wassermann (1978). Lyon: IARC. 212 pp. U.S. $45 net.

In the Foreword and Introduction to this volume, the Director of the IARC and the Editor-in-Chief attempt to set the scene for a series of publications on analytical techniques for environmental carcinogens. They stress the importance of reliable analytical information in epidemiological research and see the publication of selected analytical methods as one way of generating comparable data on levels of carcinogens.

This first volume of the series covers the determination of volatile nitrosamines in food. Although almost completely analytical in nature, the book has an extremely useful introductory chapter which summarizes current knowledge on the carcinogenicity of N-nitroso compounds. In the main part of the book there are 6 short review articles on the various aspects of nitrosamine analysis. These cover everything from taking a representative sample through extraction and clean-up to the variety of options available for the final determination. Each article is written by a person active in the field of nitrosamine analysis, and I found them very readable. They present evaluated information which otherwise would require a great deal of time and effort to assemble. This section of the book also covers the analysis of amines. nitrates and nitrites because of their potential as precursors of nitrosamines. There is a composite set of more than 250 literature references.

A section entitled "Methods of Analysis" presents a series of 11 methods which have been selected as generally representative of those shown to be reliable in IARC collaborative studies. These methods are described in great detail, and a standard format is adopted throughout the section. Topics such as principles, hazards, reagents, apparatus and procedure are covered, and each method contains references to the author's published work on the subject. Although the presentation resembles that of a cookery book, it is extremely clear and looks as if it would be ideal to follow at the bench. Each method ends with a useful schematic representation of the procedure, the address of the originating laboratory and the name of a contact person.

I would strongly recommend this short book as a starting point for anyone contemplating entering the field of nitrosamine analysis.

G. T. Steel